# Incidence and types of complications after ablative oral cancer surgery with primary microvascular free flap reconstruction

**DOI:** 10.4317/medoral.20657

**Published:** 2015-06-27

**Authors:** Johannes N. Lodders, Satyesh Parmar, Niki LM. Stienen, Timothy J. Martin, K. Hakki Karagozoglu, Martijn W. Heymans, Baljeet Nandra, Tymour Forouzanfar

**Affiliations:** 1 DDS, DDS . DDS, MD. DDS, MD, PhD. Department of Oral and Maxillofacial Surgery/Oral Pathology, Oral and Maxillofacial Surgeon, Professor and Head of department Oral and Maxillofacial Surgery/Oral Pathology; 2 PhD. Assistant professor Department of Epidemiology and Biostatistics, Department of Epidemiology and Biostatistics,VU University Medical Center/Academic Centre for Dentistry Amsterdam (ACTA),University of Amsterdam and VU University Amsterdam, De Boelelaan 1118, P.O. Box 7057,1007 MB Amsterdam, The Netherlands; 3 BChD, BMBS, BMedSCi, FDSRCS, FRCS (OMFS). BDS, MBChB, MSc, FDSRCS, FRCS (OMFS).Oral and Maxillofacial Surgeon, Head and Neck, Reconstructive Surgeon , Department of Oral and Maxillofacial Surgery, University Hospital Birmingham NHS Trust, Queen Elizabeth Hospital, Edgebaston, Birmingham, B15 2TH, United Kingdom

## Abstract

**Background:**

The aims of the study were 1) to evaluate the incidence and types of postoperative complications after ablative oral cancer surgery with primary free flap reconstruction and 2) identify prognostic variables for postoperative complications.

**Material and Methods:**

Desired data was retrieved from a computer database at the department of Oral and Maxillofacial Department, Queen Elisabeth hospital Birmingham, United Kingdom, between June 2007 and October 2012. Logistic regression was used to study relationships between preoperative variables and postoperative outcomes.

**Results:**

The study population consisted 184 patients, comprising 189 composite resections with reconstruction. Complications developed in 40.2% of the patients. Three patients (1.6%) died, 11.1% returned to the operating room, 5.3% developed donor site complications and 6.9% flap complications of which 3.2% total flap failure. In the multivariable analysis systemic complications were associated with anaesthesia time and hospital stay with red cell transfusion.

**Conclusions:**

A significant proportion of the patients with primary free flap reconstructions after oral cancer surgery develops postoperative complications. Prolonged anaesthesia time and red cell transfusion are possible predictors for systemic complications and hospital stay respectively. Preoperative screening for risk factors is advocated for patient selection and to have realistic information and expectations.

**Key words:**Free flap, complications, oral cancer, risk factors, reconstruction.

## Introduction

Oral cancer ablation can lead to considerable oro-facial defects, with inadequate aesthetics and function (1-3). Primary reconstruction of oro-facial defects with autogenous free flaps can improve function and aesthetics and thereby improve the quality of life (4,5). In experienced hands and with modern day techniques the survival rate of these free tissue grafts often exceed 90% (6-11) and is still improving due to research and innovations (8). This makes current micro vascular reconstruction very reliable, and as emphasised in the last decades, the first choice of option after major ablative cancer surgery (12,13).

Despite major advantages, free flap surgery is complex and serious postoperative morbidity with even mortality can occur. Postoperative complication rates vary between 9.3% and 64% (7,9-11,14-18). To minimize postoperative complications, authors have analysed possible prognostic factors such as demographic, anaesthetic, surgical and comorbidity variables. Some of these factors, including age (10,11), smoking (10,11), comorbidity (7,10,11,15,18), donor site (14), operating time (15,17-19) and advanced disease (19) could have a profound influence on postoperative complications. However, robust evidence is lacking that identify key variables for developing postoperative complications (7).

This study aims to retrospectively evaluate the incidence and types of postoperative complications after ablative oral cancer surgery with primary free flap reconstruction. Additionally we tried to identify variables that could have a prognostic value for postoperative complications and hospital stay.

## Material and Methods

- Data extraction

This retrospective analysis was done at the department of Oral and Maxillofacial Department, Queen Elisabeth hospital Birmingham, United Kingdom. A computer database was used to select all oral cancer surgeries with primary free flap reconstruction, between June 2007 and October 2012. Reconstructions of soft tissue as well as bony tissue or combinations were included. Resection of recurrent or second primary oral cancers were also selected.

- Study variables

By reviewing the electronic medical records, patient data was collected such as demographics, co morbidities, histopathological, anaesthetic and surgical data. Patient demographics comprised age, gender, weight, body mass index (BMI), nicotine use, alcohol use, chemotherapy and radiotherapy. The patient’s comorbidities were subdivided in different organ systems (i.e. cardiovascular, respiratory, gastrointestinal, hepatic, renal, endocrine, neurologic, autoimmune, connective tissue, prior malignant diseases, nutritional). All patients were classified according to their comorbidities in prospect using the American Society of Anaesthesiologists (ASA) classification and in retrospect using the Charlson Comorbidity index (CCI) (20).

Operating time, anaesthesia time, hospital stay, place of malignancy, donor site, primary implant placement, thromboprophylaxis and per operative red cell transfusion were collected for anaesthetic and surgical data. The period of admittance to the hospital until discharge was defined as hospital stay.

The histopathological T- and N-scores were used for anatomic staging, according to the American Joint Committee on Cancer (AJCC) staging grouping.

- Outcome variables

Postoperative complications were defined as any adverse developments that required intervention, compromised the postoperative course or when readmission to the hospital was required. A distinction was made between surgical and systemic complications. Surgical complications were defined as adverse events considering the flap, recipient site or donor site. Systemic complications were defined as medical adverse events not considering the flap, recipient site or donor site. Patients with multiple surgical or systemic complications were classified as one surgical or one systemic complication for statistical analysis.

- Statistical analysis

The SPSS Software package (version 20.0 Inc., Chicago, IL, USA) was used for statistical analysis. Univariate and multivariable relationships between surgical complications, systemic complications, hospital stay>15days and the preoperative variables were studied using binary logistic regression. Variables with a p value of <0.25 were included in the multivariable analyses. A 2 tailed p value of <0.05 was considered statistically significant and the confidence interval was set at 95%. For all missing values, analogue patient notes were retrieved from storage and searched to include missing values. The overall population average was used for missing continuous variables. Continuous variables were dichotomized if necessary, according to clinical judgement.

Ethical approval by a medical ethical commission and informed consent were not required due to the retrospective study design. All patient details were processed anonymously and not traceable to any individual. Furthermore, the data did not influence the hospital policy regarding clinical patient management.

## Results

The study population consisted of 184 patients, comprising 189 composite surgical resections with free flap reconstruction. Sixty point three percent was male and 39.7% was female. The mean age was 60.3 (standard deviation (SD) ±12.3) years and the mean BMI 25.5(SD ±5.3) kg/m2. Active nicotine use was noted in 65 (34.4%) patients, 61 (32.3%) patients never used nicotine and 63 (33.3%) patients were prior nicotine users. Forty nine (25.9%) patients reported alcohol abuse and 6 (3.2%) patients had a history with alcohol abuse. Preoperative chemotherapy was given in 2.6% of the patients, 14.8% received postoperative chemotherapy and 1.6% received both. Preoperative radiotherapy was given in 2.6% of the patients, 64.6% received postoperative radiotherapy and 7% received both.

In  Table 1, all patients’ pre-operative medical comorbidities and comorbidity indexing are shown.

**Table 1:**
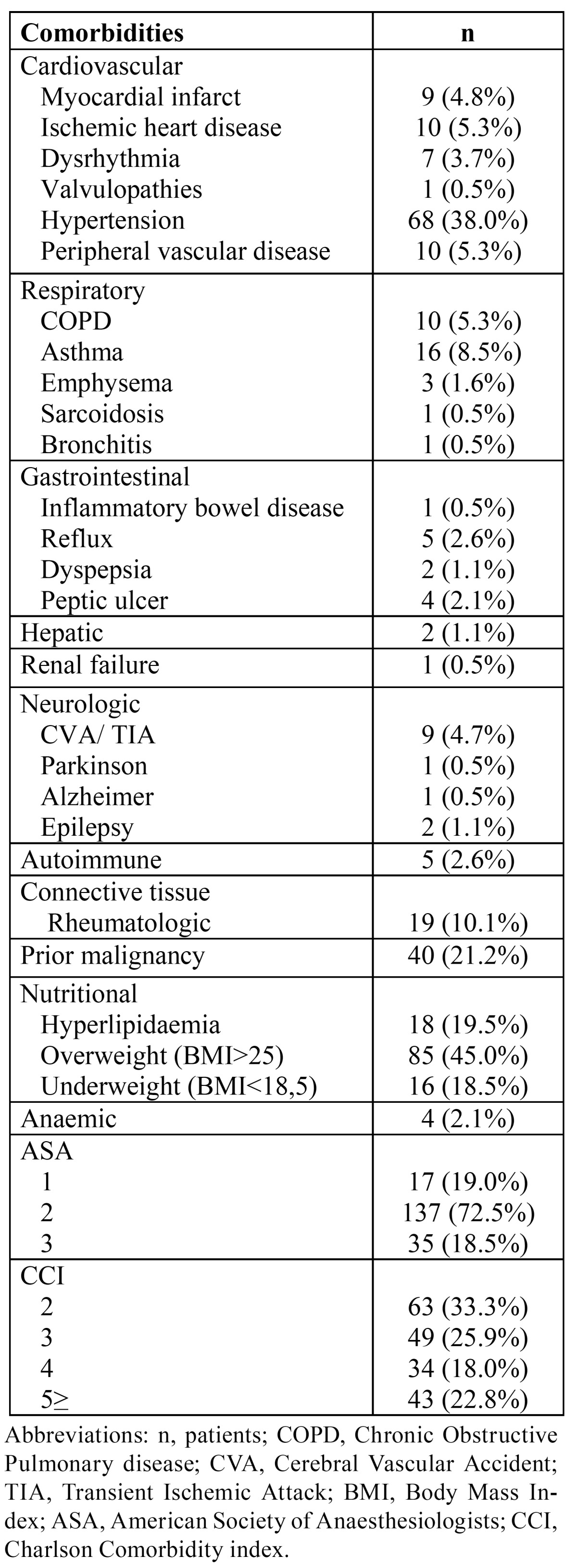
All patients’ preoperative medical comorbidities and comorbidity indexing.

**Figure 1:**
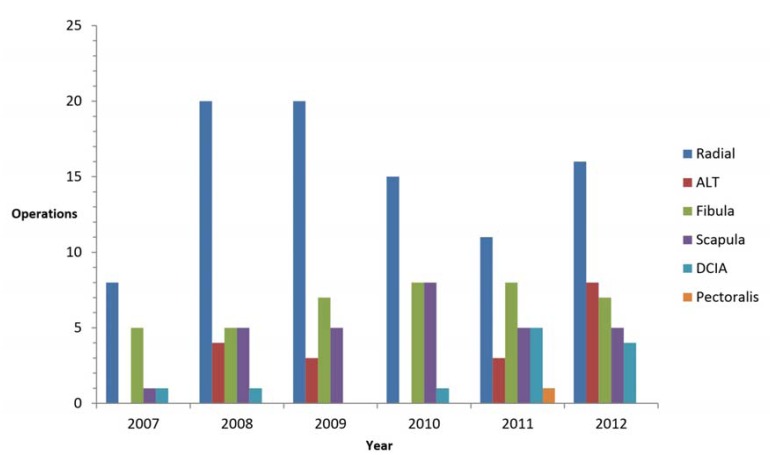
Distribution of operations according to free flap donor site by year, from June 2007 to October 2012. In 1 patient a combined reconstruction with a DCIA and ALT was performed in 2011. Abbreviations: ALT, antero lateral thigh; DCIA, deep circumflex iliac artery.

Ninety radial forearm free flaps (RFFF), 18 antero lateral thigh free flaps (ALTFF), 40 fibular free flaps (FFF), 29 scapular free flaps (SFF), 12 deep circumflex iliac artery free flaps (DCIAFF) and a pectoralis major free flap (PMFF) were used. In figure 1, all operations are plotted according to free flap donor site by year.

The anaesthesia times ranged from 366 to 959 minutes with a mean of 615.7 minutes and a mean hospital stay of 15.3 (SD ±7.0) days. One hundred and five resections could be classified as an anatomic stage IV cancer, with 89.9% of the cancers diagnosed as a squamous cell carcinoma. ( Table 2), shows all patients according to anaesthetic, surgical and histopathological variables.

**Table 2:**
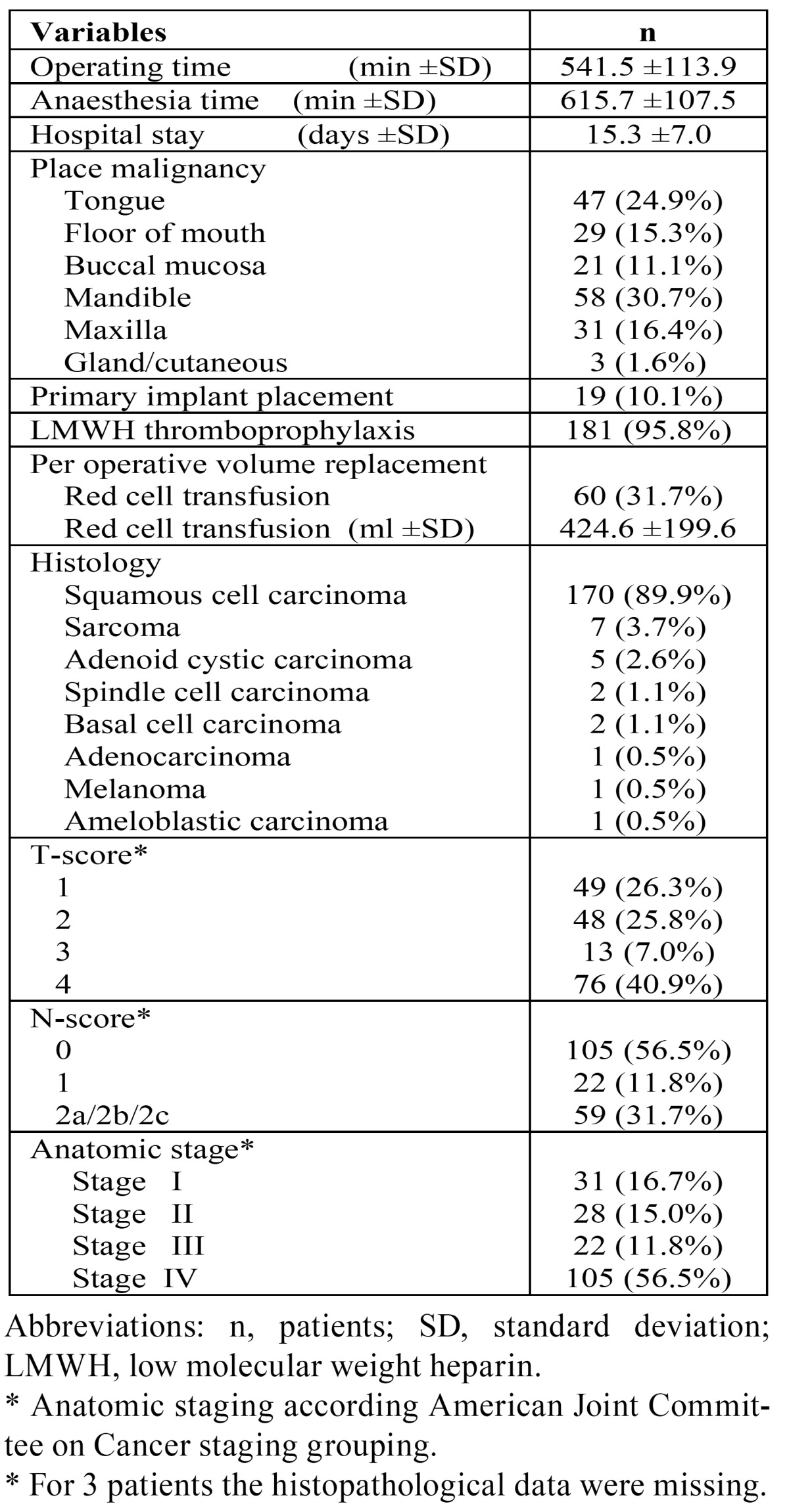
All patients’ anaesthetic, surgical and histopathological variables.

Postoperative 115 complications (98 surgical complications, 17 systemic complications) developed in 76 patients. Three patients died within 2 weeks ( Table 3). Twenty one patients had to be brought back to the operating room; debridement of total (n = 6), or partial free flap necrosis (n = 2), re-exploration of the micro vascular anastomosis (n = 5), infected/exposed plate or bone (n = 3), wound bleeding or haematoma (n = 3), abscess evacuation (n = 1) and debridement of split skin graft failure (n = 1). Donor site problems were registered in 10 patients, caused by wound infection (n = 6), wound breakdown (n = 1), wound dehiscence (n = 1), scar herniation (n = 1) and split skin graft failure (n = 1).

**Table 3:**
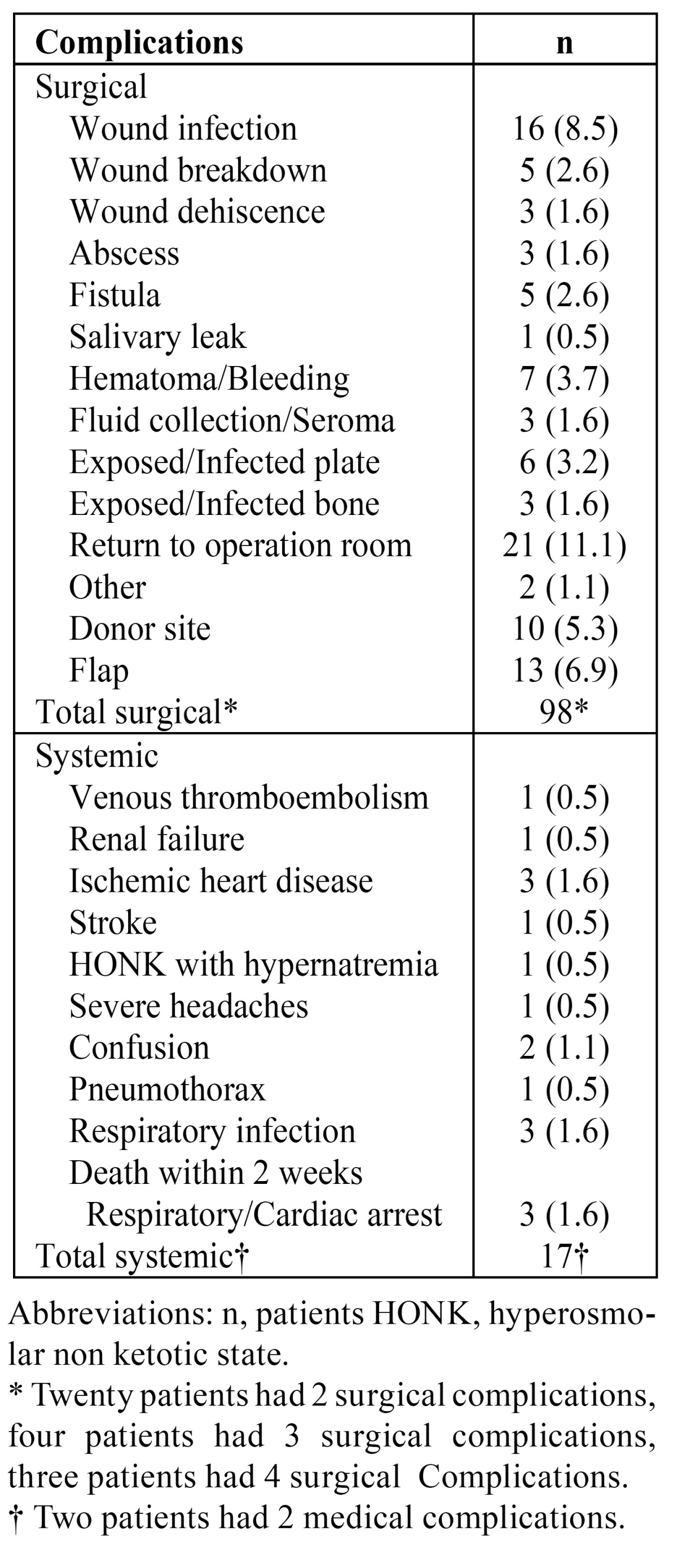
Incidences of post -operative surgical and systemic complications.

Total flap failure was diagnosed in 3 RFFFs (3.3%), 1 ALTFF (5.6%), 1 FFF (2,5%) and 1 DCIAFF (8.3%), giving an overall survival rate of 96.8%. In 2 DCIAFF the total skin paddle was loss due to necrosis. Re-exploration of the micro vascular anastomosis was necessary in 2.6% of the patients, caused by venous congestion (n = 3), anastomosis bleeding (n = 1) and an unknown cause (n = 1). All anastomosis re explorations were successful.

In the univariate analysis surgical complications were not associated with any risk variables. Associations were found between alcohol use, CCI >5, anaesthesia time, T-score ≥3 and systemic complications (*p* value = 0.04, 0.03, 0.01 and 0.01). Hospital stay>15days was related with age, preoperative chemotherapy, anaesthesia time, reconstruction type (bony), per operative red cell and T-score ≥3 (*p* value = 0.04, 0.04, 0.01, 0.00, 0.00 and 0.03) 

In the multivariable analysis none of the variables correlated with surgical complications. Associations between anaesthesia time and systemic complications remained significant, however alcohol use, CCI >5, reconstruction type (bony) and T-score ≥3 lost significance. Per operative red cell transfusion was the only variable that remained significant for hospital stay >15days in the multivariable analysis ( Table 4).

**Table 4:**
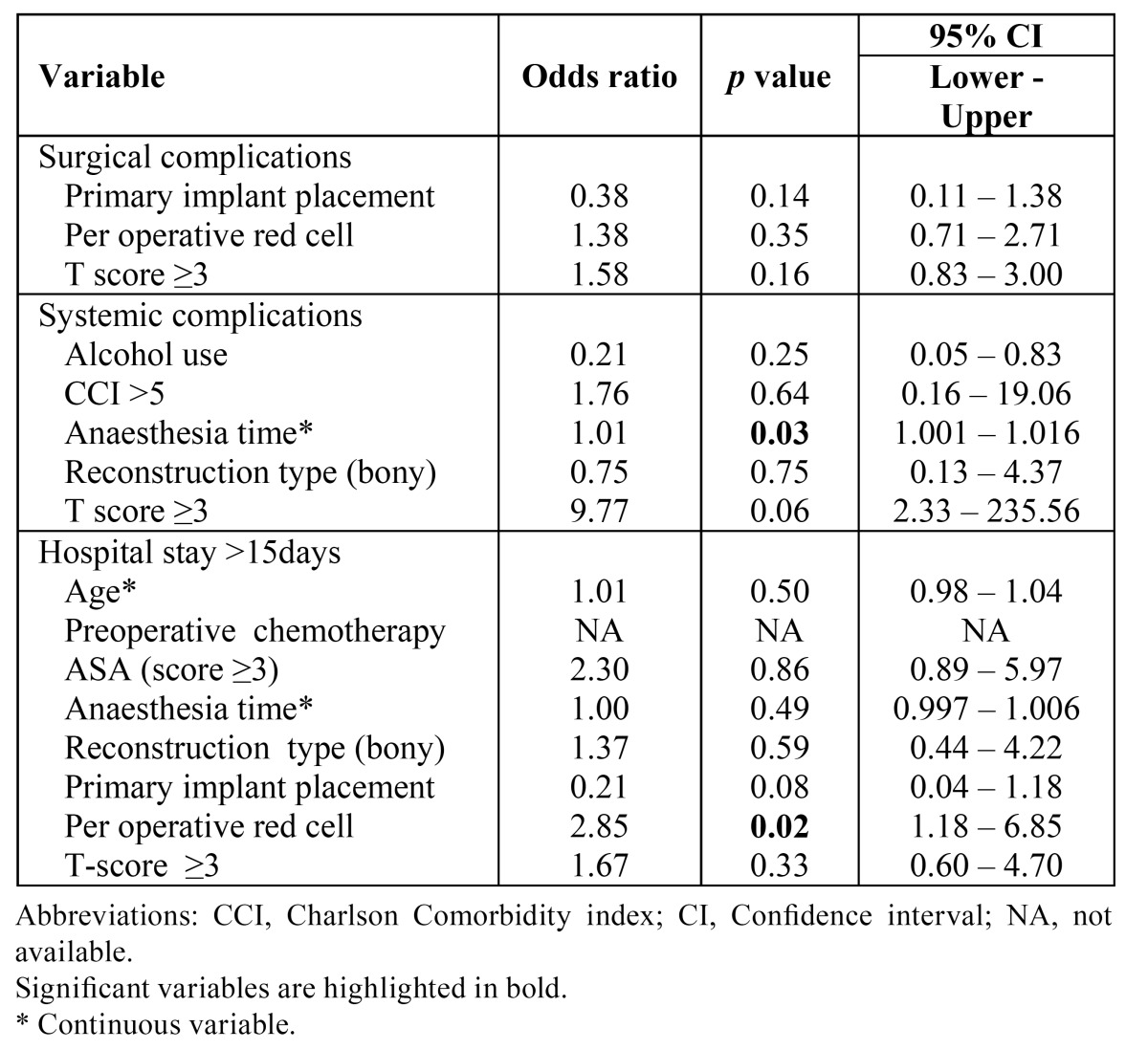
Multivariable regression analysis for surgical and systemic complications and hospital stay >15days.

## Discussion

Ablative oral cancer surgery can lead to considerable oro-facial defects, consisting soft tissue (mucosa and skin) and/or hard tissue (bone and teeth). Primary reconstruction of these defects with free flaps is a central part in head and neck surgery nowadays (12,13). High success rates (6-12,14,15), better disease control (12) and a better quality of life (4,5,12) are the motives for the popularity of free flap reconstructions. On the contrary free flap reconstructions are time and resource consuming, complex and postoperative complications are common. In rare cases mortality occurs after free flap transfers.

This retrospective study evaluated the incidence and types of postoperative complications in a well-defined cohort of 189 patients with oral cancer and primary free flap reconstruction. In total 40.2% of the patients developed postoperative complications (32.3% surgical complications and 7.9% systemic complications).

Complication rates following free flap transfers to the head and neck vary between 9.3% and 64% (7,9-11,14-18). Van Gemert *et al*. (14) retrospectively analysed 46 FFF, 22 DCIA’s and 15 RFFF for postoperative complications in patients with malignant and benignant lesions. They found that 29% of the patients developed complications in the first year after surgery. A postoperative complication rate of 53% was found by Clark *et al*. (10) in 185 free flap reconstructions, however only 40% was considered major. In a prospective study by MacMahon *et al*. (18) 192 free flap patients were studied over 27 months. They found postoperative complications in 64% of the patients and around one third were serious.

Compared to other studies our complication rates are relatively high. The variety of definitions for postoperative complications is the main reason for that. Furthermore different scoring systems for postoperative complications are used. For example distinction is made between surgical and medical (10), minor and major (15) or mild, moderate and severe (7) complications. The integration of a standardized scoring system for postoperative complications will result in efficient comparison between studies with inherent improvement of practice and quality of care (21). The Clavien-Dindo grading system (22) for postoperative complications has been adopted in 2 recent head and neck studies (18,21). Despite the fact both studies advocate its use, shortcomings regarding the unique complications in head and neck patients are acknowledged (21). Therefore future studies are needed for developing a unique classification system for postoperative complications in head and neck patients.

During this study the workhorse for intra oral soft tissue defects was the RFFF, over time the ALTFF gained more popularity. The shift can be explained by the versatility in harvesting fat, fascia or skin, low donor site complications and reliability of the ALTFF (23). Furthermore the ability to harvest the ALTFF as multiple skin paddles makes it very useful in complicated soft tissue reconstructions in the oral cavity. Other institutions confirm the increase in the use of the ALTFF over the last decade (8,23).

We found an overall flap failure rate of 3.2%, which is similar to other reports (6.2% (9), 1.6% (10), 4.3% (11), 2.4% (14), 2% (17), 3.4% (24). Compared to the most robust data currently available published by Wu *et al*. (8), almost identical results were seen. This recent retrospective single institutionary analysis transferred 2019 ALTFF, FFF, RFF and jejunal flaps to the head and neck region and reported total flap failure of 3.8%.

Predicting postoperative complications is a comprehensive matter, because different variables cannot predict all forms of complications (10). Therefore key factors are difficult to find, however certain variables seem to have predictive value, such as age (10,11), smoking (10,11), comorbidity (7,10,11,15,18), donor site (14), operating time (15,17-19) and advanced disease (19)

No risk factors were identified in the univariate or multivariable analysis for surgical complications in these series. We found correlations between anaesthesia time and systemic complications in the multivariable analysis. Other studies (15,17,18) also associated operating time with postoperative complications. Pohlenz *et al*. (19) even associated increased operating time with postoperative mortality. Therefore aggressive surgery should be indicated with caution in complex head and neck patients with an expected prolonged operating time.

Predictive values for smoking, pre-operative radiotherapy and comorbidity could not be found, despite the negative influence these variables have on the healing process (25). The ASA classification and CCI were not associated with postoperative complications. Nevertheless medical comorbidities are the most established predictors for postoperative complications (7,10,11,15) and therefore extensive preoperative medical screening with optimization of the patients’ comorbidities should be a vital part in treatment planning.

Several factors were associated with hospital stay >15days. Only per operative red cell transfusion (OR 2.85) was significantly correlated in the multivariable analysis. A handful (10,11) of authors address the importance for optimal fluid management during surgery. Haughley *et al*. (11) correlated, similar to our findings, red cell transfusion to hospital stay in a retrospective analysis of 141 free flap reconstructions.

The retrospective nature of this study can be a flawed method for analysing data, because the data rely on adequate record keeping. Therefore important exposure variables were not retrieved, such as the use of tracheostomy. The importance of record keeping is reflected in the aberrant low incidence of respiratory problems (1.6%), compared to other studies (10-18%) (7,10,11,16) and the main reason why variables did not reach statistical significance in the multivariable analysis.

A significant proportion of the patients with primary free flap reconstructions after oral cancer surgery develops postoperative complications. Prolonged anaesthesia time and red cell transfusion are possible predictors for systemic complications and hospital stay respectively. These and other risk factors are relatively unchangeable, so little influence can be exerted on the patient’s outcome. Therefore preoperative screening for risk factors is advocated for patient selection and to have realistic information and expectations.
